# Vision transformer-based uncertainty quantification for triaging skin lesions: a probabilistic framework for automated biopsy recommendation

**DOI:** 10.3389/fbioe.2026.1859844

**Published:** 2026-06-29

**Authors:** Jafaridarabjerdi Mahin, Lin Li

**Affiliations:** 1 Faculty of Medicine, Dalian University of Technology, Dalian, Liaoning, China; 2 Central Hospital of Dalian University of Technology, Dalian, Liaoning, China; 3 School of Basic Medicine, Faculty of Medicine, Dalian University of Technology, Dalian, Liaoning, China

**Keywords:** evidential deep learning, multimodal fusion, skin lesion triage, Swin Transformer, uncertainty quantification

## Abstract

**Introduction:**

Automated skin lesion detection systems based on deep learning have been found to have significant potential in melanoma triage, but have not been widely used in clinical practice because they use visual features to make predictions (without patient history) and make definitive predictions with false overconfidence. This paper introduces a new probabilistic model, Evidential Deep Learning and Vision Transformer architecture, that produces safer biopsy suggestions through uncertainty quantification.

**Methods:**

The model was trained and tested using the ISIC composite database (10,010 records). The hierarchical Swin Transformer architecture is utilized in the proposed framework to extract complex features of the dermoscopic images. To combine multimodal data, a cross-attention system is trained, which learns the fine-grained correlations between visual features and patient clinical data (age, gender and lesion location). Lastly, the system, with the Dirichlet distribution in the decision layer, not only predicts the need to order a biopsy, but also produces a quantifiable uncertainty estimate that can be used to refer suspicious and out-of-distribution cases to a specialist.

**Results:**

The results of the evaluation indicate that the suggested framework had an accuracy of 92.398% and an area under the operating characteristic curve (AUROC) of 0.924. More importantly, the incorporation of evidential learning resulted in a reduction in the expected calibration error (ECE) by 0.031 and improved the model’s sensitivity in detecting lesions requiring biopsy to 92.37%. It was also able to recognize 89.5% of low-quality or ambiguous images and tag them as in need of expert review.

**Discussion:**

The suggested multimodal and uncertainty-aware system not only enhances diagnostic accuracy by infusing clinical context, but also decreases human and systematic error in dermatology triage by offering a computational safety layer. This system is an effective step towards implementing Trustworthy AI in healthcare workflows.

## 1 Introduction

One of the most widespread and hazardous types of cancer in the world is skin cancer and melanoma, in particular, and early diagnosis is a key factor in the effectiveness of treatment and a higher survival rate among patients (Siegel et al., 2024). The accuracy of diagnosis has greatly increased with the use of clinical examination in the form of dermoscopy images, though this still depends greatly on the experience and the expertise of the physician and is subject to human error (Zbrzezny and Krzywicki, 2025). Recently, remarkable progress in artificial intelligence and deep learning, particularly, convolutional neural networks (CNN) and vision transformers, has created new opportunities in the automated study of skin lesions (Shamshad et al., 2023). Even with these developments, the overriding question of the present study is how to make an intelligent triage system that is not only highly accurate in analyzing the lesions, but capable of giving biopsy advice to the physician, considering the level of confidence of the model and the clinical metadata of the patient, in a manner that is medically acceptable.

Regardless of the successes, there are two significant research gaps in the current automated diagnosis systems. First, these models consider only the visual qualities of images and ignore critical clinical data about the patient (age, gender, the location of the lesion on the anatomy) that are important in the decision-making of clinicians in the real world (Wen et al., 2022). Second, and possibly more crucially, the general use of deep learning models based on conventional activation functions (like Softmax) contributes to overconfidence in clear predictions (Gawlikowski et al., 2023). These models cannot differentiate between mistakes due to low quality of images and model ignorance. In medical practice involving sensitive cases, the amount of uncertainty should not be neglected as it may result in the occurrence of errors that cannot be corrected, like the failure to refer the cases of suspicion to a specialist or the unnecessary biopsy (Begoli et al., 2019). To address these gaps, the current research presents a new probabilistic system, which relies on visual transformers and evidential deep learning to determine the uncertainty of skin lesion triage. Although some efforts have started to tackle these challenges in 2025 and 2026 with Bayesian recalibration (Wang et al., 2026) and conformal prediction sets (Zoravar et al., 2025), many continue to face the “trilemma” of high computational costs, inference latency, and the black-box nature of foundation models (Lu et al., 2026). We are contributing to this landscape by providing a framework that is evidential and is based on a single pass, rather than stochastic sampling, that integrates clinical context without the overhead of stochastic sampling.

The hierarchical Swin Transformer architecture is employed in this method in order to precisely extract visual features at various scales. The key novelty of this model is the smart and multi-modal integration of images with clinical data with the Cross-Attention mechanism (Xu et al., 2023). Furthermore, by replacing the traditional decision layers with an evidential learning approach based on the Dirichlet Distribution, the proposed model is able to not only predict the need for biopsy, but also calculate an explicit “uncertainty score” (Han et al., 2024). This will take care of the cases where the patient is not clear-cut, the system will simply send the patient to a specialist to do further research instead of delivering a high-certainty false verdict. Based on this, the key contributions and accomplishments of this paper are as follows:

- Introducing a unified model that incorporates the multifaceted nature of dermoscopic images with the clinical data of the patient to enhance the accuracy of the diagnosis in complicated cases and lesions of this type.
- Creation of a probabilistic decision layer, which simulates the outputs of neural networks as evidence and is capable of safely identifying out-of-distribution data and low-quality images.
- Mapping the multi-class disease classification problem to a dual triage system that can be used with dermatology protocols and identifying a referral mechanism to a specialist depending on the uncertainty threshold.
- Application and testing of the framework on a valid dataset and demonstration of its excellence compared to deterministic models in the view of calibration and medical reliability.

The remainder of this paper will be structured as follows: Section 2 will provide a review of the research background and associated works in the area of automated skin lesion diagnosis and uncertainty modeling. Section 3 describes in detail the research methodology, the structure of the dataset, and the details of the proposed multi-modal architecture. Section 4 provides and discusses the outcomes of the experiment, qualitative and quantitative assessment and compares the performance of the model with other existing methods. Lastly, Section 5 is the conclusion of the research and recommendations on future research.

## 2 Related works

Automated dermatological diagnosis has rapidly changed its appearance and instead of being simply a classification process, it has developed into complex systems that strive to emulate clinical reasoning. Much of the early work was devoted to improving predictive power by nature-inspired optimization and meta-heuristic algorithms to optimize conventional machine learning and early deep learning models.

### 2.1 Optimization and meta-heuristic approaches

Initial efforts to enhance the performance of skin cancer detection tended to be based on the concept of mixing neural networks with evolutionary algorithms to explore high-dimensional parameter space. Han et al. (2024) proposed an integration of Echo State Networks (ESN) with a “Seasons Optimization” algorithm. This was done by simulating the seasonal adaptations in nature to fine-tune the recurrent network parameters in order to enhance the speed of pattern recognition. Nevertheless, ESNs can be computationally efficient, but they can have problems with the intricate spatial hierarchies of dermoscopic images. On the same note, Bi et al. (2021) concentrated on the feature selection bottleneck by integrating Support Vector Machines (SVMs) with meta-heuristic search to determine the most discriminative features of the lesions. This method was useful in dimensionality reduction but not as powerful as the end-to-end representation of deep learning.

Continuing the pattern, Rajendran and Shanmugam (2024) proposed a multi-stage pipeline, which uses U-Net to segment lesions, MobileNet to extract features, and Cat Swarm Optimization (CSO) to tune the hyperparameters of a Gated Recurrent Unit (GRU) classifier. Although extensive, the dependence on several disjoint parts may result in the erroneous spread throughout the pipeline. Mohakud and Dash (2022) tried to stabilize CNNs training by using the Gray Wolf Optimization to establish the best set of convolutional filters. Although this improved the filter properties, the model was still a deterministic black box with no mechanism to self-assess itself. More recently, Zhang et al. (2024) applied an Orca Predation Algorithm to optimize GRU networks, in particular, the temporal or sequential nuances of image data. Although these optimization-based models achieve high validation results, they are more concerned with the best-case-prediction and do not take into account the risk of overconfident misclassification.

### 2.2 Architectural innovations and feature engineering

With the maturation of deep learning, studies began to focus on more specific architectures that could help to capture the details of skin pathologies. The approach by Mushtaq and Singh (2024) to common clinical noise involved a preprocessing phase of hair removal with an ensemble of VGG-16 models. Initially training the same architecture several times and averaging the outcomes they aimed to enhance generalization, but ensemble methods are typically computationally infeasible in real-time mobile applications. Conversely, Nawaz et al. (2025) addressed the long-tail problem of the distribution associated with medical data by introducing a tailored FCDS-CNN. They added class weighting and vigorous data augmentation, making the model learn the properties of rare yet deadly conditions such as melanoma, but the system did not have a mechanism of alerting when an image was too low-quality to make a safe prediction.

The latest hybrid models have tried to integrate the local feature extraction of CNNs with the global context of Vision Transformers. Pacal et al. (2025), Ozdemir and Pacal (2025) investigated designs based on MetaFormer and ConvNeXtV2 blocks with focal self-attention mechanisms. These focal systems focus on suppressing background noise and directing the model to pay attention to diagnostically important areas. Equally, Claret et al. (2024) incorporated Discrete Wavelet Transformation (DWT) into the CNN pipeline to obtain multi-scale spectral features that the traditional convolutions tend to overlook. Although these architectural enhancements greatly increase accuracy, they do not necessarily offer a degree of confidence and are hard to rely on in high-stakes triage cases.

Continuing the trend toward medically-inspired architectures, DermViT (Zhang et al., 2025) proposed a Dermoscopic Context Pyramid (DCP) and Hierarchical Attention (DHA) mechanism inspired by the multi-scale observation paradigm adopted by human dermatologists. In contrast to the standard ViT-Base, DermViT aims to improve feature extraction and feature parameter reduction, but it does not include a built-in probabilistic layer to capture the uncertainty of the diagnosis in ambiguous or out-of-distribution cases.

### 2.3 Uncertainty quantification and multimodal integration

To shift to Trustworthy AI, models will be needed that are capable of measuring their own ignorance. The DUNEScan system by Mazoure et al. (2022) estimates the uncertainty by computing the variance of a set of different CNN frameworks, including ResNet and Inception. Although this can give a heuristic to diagnostic ambiguity, it is based on the agreement of many models, instead of a grounded probabilistic framework in one network. Other scientists have investigated the use of image data together with ensemble methods and patient history to reflect the holistic vision of dermatologists. Gamil et al. (2024) applied Principal Component Analysis (PCA) in dimensionality reduction and AdaBoost to reinforce the EfficientNet features classification.

In order to combine visual features and clinical context, Kanchana et al. (2024) applied the EfficientNet series and used transfer learning and incorporated patient-specific data. Despite the fact that this multimodal strategy enhanced performance, the combination was usually basic, and there was no system to study the fine-grained relationships between tabular and visual data. Lastly, pipelines with high-performance such as the EfficientNetV2L-LightGBM ensemble suggested by Swapno et al. (2025) have shown close-to-perfect accuracy on standard benchmarks. Nonetheless, these models do not have an evidential structure and hence are unable to differentiate between an easy correct prediction and a lucky guess on an out of distribution sample.

Although less studied in dermatology triage, Evidential Deep Learning (EDL) has proven to be very successful in other high-stakes scientific settings for quantifying ‘known unknowns’ without the computational cost of ensembles or Bayesian sampling. For example, Soleimany et al. (2021) used EDL for guided molecular property prediction, showing that evidential uncertainties help to make calibrated predictions with uncertainty that is directly related to error, allowing more sample-efficient active learning. Likewise, in the drug discovery field, Zhao et al. (2025) proposed EviDTI, a framework that leverages EDL to generate reliable confidence scores for drug-target interaction prediction, which improves the robustness of the model in the face of novel or unseen drugs. Moreover, Wang et al. (2023) employed an uncertainty-guided approach to speed up the screening of CYP3A4 inhibitors, where the evidential uncertainty is utilized as a measure of reliability for prioritizing high confidence candidates, thereby minimizing the number of false positives in virtual screening. The versatility of EDL as a strong computational safety layer in predictive modeling is demonstrated in these applications.

In 2025 and 2026, the trend towards “conscious” models that are aware of their own limitations has been emphasized for the shift towards Trustworthy AI. Wang et al. (2026) proposed DermaCalibra, using the Bayesian uncertainty estimation using Monte Carlo dropout to adapt the weights of focal loss, which effectively prioritizes underrepresented classes. Bayesian methods offer good calibration, but require multiple forward passes, which can be problematic for real-time triage. Conformal prediction, on the other hand, has become a well-founded alternative for safety guarantees. Zoravar et al. (2025) proposed an ensemble of Vision Transformers (CE-ViTs) based on conformal learning to achieve high coverage rates, thereby enhancing the probability that the true label is included in the prediction set. Likewise, Bhattacharyya et al. (2025) used conformal analysis to Google’s DermFoundation model to measure predictive fairness across age and ethnicity, with uncertainty as a measure of mitigating algorithmic bias.

Recently, the fusion of Vision-Language Models (VLMs) has brought a new paradigm to multimodal fusion. SkinCLIP-VL (Lu et al., 2026) adopts a ‘frozen perception, adaptive reasoning’ paradigm where visual regions are matched with clinical semantics via a Consistency-aware Focal Alignment (CFA) loss. These VLMs offer rationales that can be explained, but they are very costly in terms of computing resources. A brief summary of the related studies is presented in Table 1. We propose a framework that differs from these sampling based (Bayesian) and heavy-backbone (VLM) approaches, using EDL. We directly model evidence in the Dirichlet distribution parameters to obtain deterministic, single-pass uncertainty quantification with the granularity of Bayesian methods and the computational efficiency needed for quick clinical triage.

**TABLE 1:** Summary of the related studies.

Methodology category	References	Key limitations addressed by this work
Meta-heuristic optimization	Han et al. (2024), Bi et al. (2021), Rajendran and Shanmugam (2024), Mohakud and Dash (2022), Zhang et al. (2024)	Focuses on parameter tuning but lacks intrinsic uncertainty estimation
Medically-driven architectures	Mushtaq and Singh (2024), Pacal et al. (2025), Ozdemir and Pacal (2025), Zhang et al. (2025)	Mimics physician paradigms for high accuracy but lacks probabilistic confidence layers
Bayesian & ensemble UQ	Wang et al. (2026), Zoravar et al. (2025), Mazoure et al. (2022)	High computational cost due to stochastic sampling or multiple forward passes
Foundation models and VLMs	Lu et al. (2026), Bhattacharyya et al. (2025)	Heavy resource requirements; often lacks task-specific cross-attention for tabular metadata
Foundational EDL in science	Soleimany et al. (2021), Zhao et al. (2025), Wang et al. (2023)	Demonstrates the utility of EDL in chemistry and drug discovery but lacks application to image-text fusion
Proposed framework	This Work	Achieves single-pass, sampling-free UQ with context-aware cross-attention for efficient clinical triage

## 3 Research methodology

This section, first explains the structure of the dataset employed in this research and then, details the steps of the proposed architecture.

### 3.1 Dataset

In this study, a comprehensive and multi-modal dataset was used to train and evaluate the proposed framework. This dataset was extracted by combining dermoscopic images and clinical information of patients from reliable and standardized databases, in particular the International Skin Imaging Archive (ISIC Archive - including HAM10000 data) (Tschandl et al., 2018). The final dataset consists of 10,010 unique records covering a wide range of benign and malignant skin lesions in different populations. Each record in this dataset is a combination of a high-quality dermoscopic image and clinical metadata related to the patient, which provides the necessary platform for training a multi-modal and context-aware triage model. Table 2 presents the grouping of features in this dataset.

**TABLE 2:** Dataset specifications.

Feature group	Features included	Data type	Description
Image data	Dermoscopic image	3D tensor (RGB)	High-resolution dermoscopy images of skin lesions, capturing morphological structures and color patterns
Clinical demographics	Age, sex	Numerical/Categorical	Patient’s age (in years) and biological sex (Male/Female/Unknown)
Anatomical metadata	Lesion localization	Categorical	Specific body part where the lesion is located (e.g., face, lower extremity, torso, back)
Diagnostic meta-labels	Diagnosis, conf. method	Categorical	The specific disease class (e.g., Melanoma, Nevus) and how it was confirmed (e.g., Histopathology, Consensus)

To prepare the data for feeding into the Vision Transformer (ViT) architecture, extensive preprocessing of the image and tabular data was performed. All images were resized to the standard dimensions of the transformer base grid (224 × 224 pixels) and pixel intensity values ​​were normalized to accelerate model convergence.

Since real-world clinical data always face the challenge of missing information, missing values in clinical features such as age were handled using Mean Imputation, while missing values for categorical features like lesion location were handled using Mode Imputation (assigning the most frequent category) or designated as a separate “Unknown” class to maintain data integrity. In addition, the inherent challenge of class imbalance in medical data—where benign samples far outnumber malignant samples—required intervention. To avoid biasing the model towards the majority classes, combined data augmentation techniques such as random rotation, scaling, and targeted brightness adjustments were used for the minority classes.

The target variable in this study for training the probabilistic framework is defined as “Biopsy Recommendation,” which acts as a binary classification variable. To accurately determine this variable, the initial multiclass diagnostic labels (e.g., melanoma, basal cell carcinoma, melanocytic nevus, etc.) were mapped into two operational decision classes based on clinical guidelines and standard dermatology triage protocols:

1. Biopsy required (1954 samples): including all confirmed malignant lesions and highly suspicious or precancerous lesions.
2. No biopsy required (8056 samples): lesions assessed as benign with high certainty.

The gold standard (Ground Truth) for the final validation of malignant classes has been exclusively the definitive results from histopathology tests, while benign lesion labels have generally been validated through expert consensus. This labeling strategy ensures that the model predictions are directly aligned with the clinical workflow of experts.

### 3.2 Proposed framework

**FIGURE 1:**
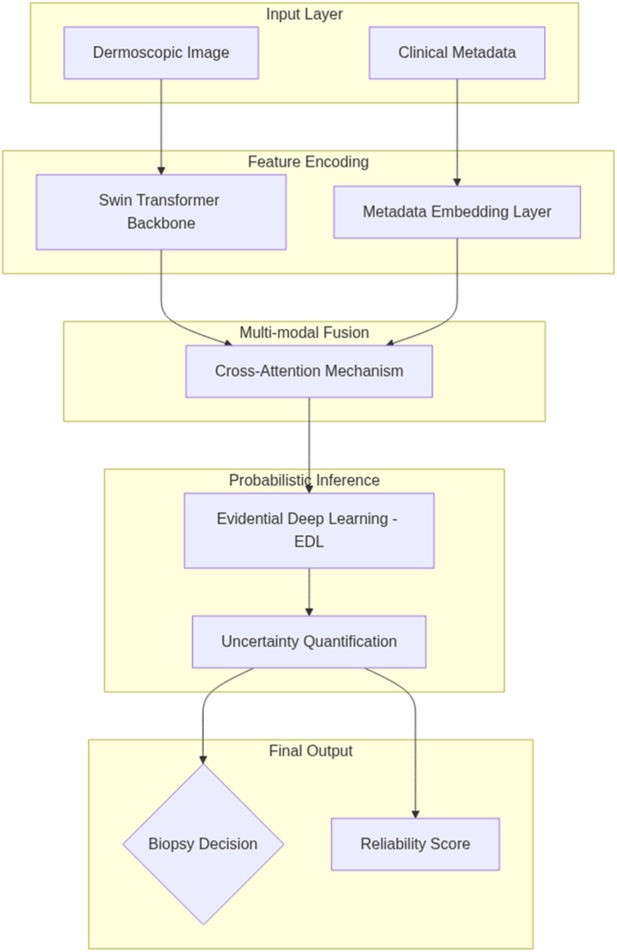
Overview of the proposed architecture for uncertainty-aware triage of skin lesions.

Traditional automated skin lesion detection systems, despite their high accuracy, often face two major challenges: first, ignoring the patient’s clinical information (such as age and lesion location) that are crucial in the physician’s final diagnosis; and second, providing deterministic predictions without considering the model’s confidence level, which can lead to dangerous medical errors in doubtful cases. Our proposed framework in this study aims to fill this gap by using an uncertainty-aware multi-modal approach. By combining the power of hierarchical feature extraction in visual transformers and evidential modeling, this method provides a system that not only recognizes the need for biopsy, but also provides a quantifiable measure of predictive confidence to support clinical decision-making. The main steps of the proposed methodology are as follows (Figure 1):

1. Data Pre-processing and Feature Encoding
2. Hierarchical Visual Feature Extraction
3. Attention-based Multi-modal Fusion
4. Evidential UQ and Classification

In the first step, input data including images and text features (such as age and gender) are fed into the encoding layers. The Swin Transformer was chosen as the core of visual feature extraction because, unlike standard transformers, this architecture, using Shifted Windows, has an excellent ability to analyze lesions with variable dimensions and irregular textures, which is crucial for accurate melanoma diagnosis. At the same time, the clinical data is mapped to a space with the same dimensions as the image, ready for fusion.

Next, to overcome the limitations of simple methods (such as concatenation), a cross-attention mechanism is used. This technique allows the model to intelligently determine which clinical feature (e.g., patient age) is most closely related to which part of the image (e.g., pigment patterns). This multi-faceted approach significantly increases the accuracy of diagnosis in cases where the image alone is not informative.

Finally, in the decision layer, instead of using the traditional Softmax function that only provides class probabilities, an Evidential Deep Learning approach is used. This choice is made so that the model can distinguish between “suspicion due to similarity of two diseases” and “suspicion due to poor image quality or lack of data”. The final output of the system not only provides a biopsy recommendation, but also produces an Uncertainty Score, which acts as a safety layer in healthcare settings, referring suspicious cases to a specialist for final review.

#### 3.2.1 Data pre-processing and feature encoding

The data preparation process is performed in two parallel paths for image data and clinical metadata to ensure that the model extracts the most information from both sources.

##### 3.2.1.1 Visual pipeline

The raw dermoscopy images have different dimensions. To maintain compatibility with the input structure of the transformer, all images are first resized to H×W=224×224 . Then, a normalization operation is performed based on the mean ( μ ) and standard deviation ( σ ) of the dataset to ensure numerical stability during training using Equation 1:

$$I_{norm}=\frac{I_{raw}-\mu }{\sigma }$$ (1)

In the visual encoding step, the image is divided into discrete patches. Unlike the classic ViT, in the proposed Swin Transformer architecture, the image is divided into patches of size 4×4 and each patch is mapped to a feature vector. If $I\in R^{H\times W\times 3}$ is the input image, after the Patch Partitioning step, a set of local features is extracted, which will be the basis for hierarchical analyses in the following steps.

##### 3.2.1.2 Clinical metadata encoding path

The clinical data introduced in Section 3.1 include two types of variables: numeric (such as age) and categorical (such as gender and lesion location).

1. Numeric variables: To prevent features with large amplitude from dominating other features, the age variable is mapped to the interval [0, 1] using the Min-Max Scaling method in Equation 2:

$$x_{age}^{\prime }=\frac{x_{age}-\min\left(age\right)}{\max\left(age\right)-\min\left(age\right)}$$ (2)

2. Categorical variables: Instead of using One-hot Encoding which results in sparse vectors, we use Embedding Layers. This layer maps each category to a dense vector in the $R^{d}$ space where semantic relationships between categories (e.g., anatomical proximity of lesion sites) can be learned. The process of generating the clinical feature vector ( $z_{meta}$ ) is expressed as Equation 3:

$$z_{meta}=MLP\left(Concat\left(e_{loc},e_{sex},x_{age}^{\prime }\right)\right)$$ (3)

where e are the vectors resulting from the embedding and MLP is a multilayer perceptron network that is responsible for matching the dimensions of the clinical features with the dimensions of the features extracted from the transformer ( D ). This dimensional matching is necessary for the correct operation of the cross-attention mechanism in the fusion stage.

Finally, the output of this section consists of two sets of vectors: a visual feature map rich in morphological details and a clinical feature vector representing the patient’s context. This infrastructure enables the model to analyze the complex relationships between the appearance of the lesion and the patient’s clinical condition in subsequent steps.

#### 3.2.2 Hierarchical visual feature extraction

After the preprocessing step, the $I_{norm}$ image is fed into the main body of the model. Unlike standard visual transformers that use fixed-size patches and constant resolution across all layers, in this study, the Swin Transformer architecture (Liu et al., 2021) is used as the backbone of feature extraction. This choice is due to the nature of skin lesions; some diagnostic features such as “pigmentation networks” are very subtle and local, while features such as “asymmetry” encompass the entire lesion structure.

##### 3.2.2.1 Hierarchical structure and patch merging

**FIGURE 2:**
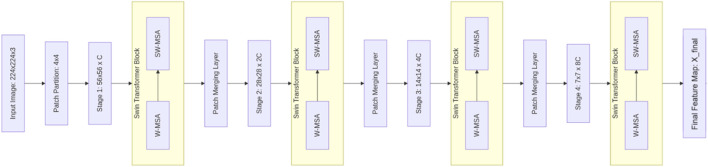
Hierarchical architecture of the visual transformer with a sliding window mechanism (Swin Transformer).

The hierarchical architecture of the visual transformer with a sliding window mechanism in the employed swin transformer is illustrated in Figure 2. The proposed model consists of four main stages. At each stage, the spatial resolution of the image is reduced and the channel dimension of the features is increased using patch merging layers. This process works similarly to CNNs and creates a hierarchical representation:

1. In the first step, the feature map dimensions are $\frac{H}{4}\times \frac{W}{4}\times C$ .
2. In the next steps, the resolution is reduced to $\frac{H}{8}\times \frac{W}{8}$ , $\frac{H}{16}\times \frac{W}{16}$ , and finally $\frac{H}{32}\times \frac{W}{32}$ , respectively, while the number of channels is doubled. In these four hierarchical levels, the Swin Transformer blocks have 3, 6, 12 and 24 heads, respectively. This network is composed of $\left[2,2,6,2\right]$ successive transformer blocks, which is able to extract increasingly complex and global multi-scale textural irregularities of skin lesions.

##### 3.2.2.2 Shifted window self-attention mechanism

The main innovation in this section is the use of the shared attention mechanism in local windows (W-MSA) and shifting windows (SW-MSA). To reduce the computational complexity, the image is divided into non-overlapping windows of size M×M (here 7×7 ). In the first layer, attention is calculated only within each window by Equation 4:

$$Attention\left(Q,K,V\right)=Softmax\left(\frac{QK^{T}}{\sqrt{d}}+B\right)V$$ (4)

where B is the relative position bias matrix to preserve spatial information. In the next layer, the window boundaries are shifted to establish communication between adjacent windows. This shifting causes the edge pixels of each window in the previous stage to interact with the neighboring pixels in the new stage, which leads to the extraction of continuous structural features from the lesion.

At the end of the fourth step, the final feature map $X_{final}\in R^{\frac{H}{32}\times \frac{W}{32}\times D}$ is preserved. Instead of immediate global pooling, this spatial feature map is passed to the fusion module to allow for localized cross-modal interaction. This hierarchical structure ensures that the model is sensitive to both fine textural details and geometric features of the entire lesion.

#### 3.2.3 Attention-based multi-modal fusion

The integration of heterogeneous data (image and text) is one of the main challenges in multimodal systems. Traditional methods such as simple concatenation assume that all features have the same importance and ignore the interrelationships between the image and the patient’s clinical context. In this study, we use the Cross-Attention mechanism so that the model can focus on or modulate the influence of specific parts of the visual features based on the patient’s clinical information. In this structure, the clinical feature vector ( $z_{meta}$ ) acts as a ‘Query’ and interacts with the spatial feature map ( $X_{final}$ ), which acts as the “Key” and “Value” (Equation 5). This ensures that clinical context can ‘attend’ to specific spatial regions of the lesion before the final global representation is formed.

$$Q=z_{meta}\;W_{Q},$$ (5)

$$K=z_{vis}\;W_{K},$$

$$V=z_{vis}\;W_{V}$$

In the above equations Q , K , and V represent the Query, Key, and Value matrices, respectively. $W_{Q}$ , $W_{K}$ , and $W_{V}$ are learnable weight matrices that are optimized during the training process to map the relationships between two data features.

Then, the output of the cross-attention mechanism ( A ), which represents the reweighted visual features based on the clinical context, is calculated through Equation 6 (Soydaner, 2022):

$$A=Softmax\left(\frac{QK^{T}}{\sqrt{d_{k}}}\right)V$$ (6)

Here, $d_{k}$ is the key dimension, which is used as a scaling factor to prevent the inner product values from becoming too large and saturating the Softmax function. This equation allows the model to pay more attention to certain visual features that are indicative of malignancy at older ages (such as regression patterns in melanoma).

Finally, to maintain training stability and exploit the original image features, a residual connection and layer normalization-as formulated in Equation 7 are used to produce the final fusion vector ( $z_{fused}$ ):

$$z_{fused}=LayerNorm\left(z_{vis}+A\right)$$ (7)

The resulting vector, $z_{fused}$ , is a unified representation that not only includes the visual features of the lesion, but is also “contextualized” by the patient’s personal characteristics. This final vector is then sent as input to the evidence classification and uncertainty quantification layer. Using this attention-based approach significantly increases the model’s accuracy in distinguishing lesions that have similar appearances but different clinical backgrounds.

#### 3.2.4 Evidential UQ and classification

**FIGURE 3:**
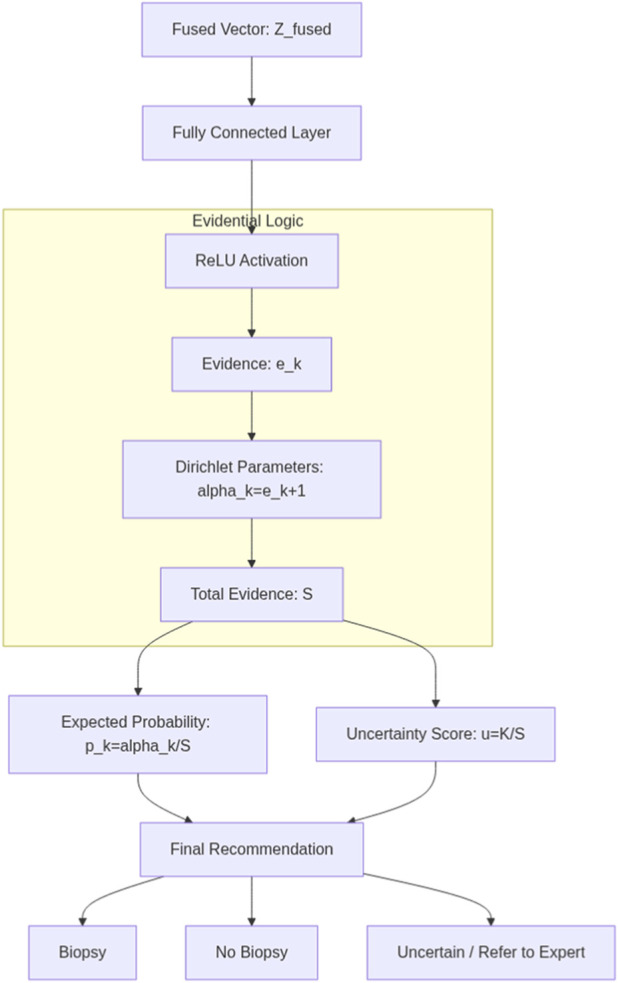
Evidence layer structure for extracting the combined probability of malignancy and uncertainty score.

Most deep models in skin lesion detection use the Softmax function, which leads to overconfidence estimates, even when the input image is poor quality or outside the training distribution. In this study, we use an evidential deep learning approach that treats the network output not as probabilities, but as “evidence” for a Dirichlet Distribution (Figure 3). In this step, the vector $z_{fused}$ is sent to a Fully Connected Layer formulated by Equation 8 to extract the evidence values for each class ( $e_{k}$ ). To ensure that the evidence is non-negative, the ReLU activation function is used:

$$e_{k}=f\left(w_{k}\cdot \;z_{fused}+b_{k}\right)$$ (8)

In this equation, $e_{k}$ is the amount of evidence assigned to class k (e.g., need for biopsy or not). According to the theory of subjective logic, this evidence determines the parameters of the Dirichlet distribution as $\alpha_{k}=e_{k}+1$ . The total amount of evidence in the system, known as the “strength of evidence”, is represented by S and is given by Equation 9:

$$S=\sum_{k=1}^{K}\alpha_{k}$$ (9)

where K is the number of target classes. Accordingly, the uncertainty of the model, denoted by u , is calculated through Equation 10:

$$u=\frac{K}{S}$$ (10)

This variable u is a number between 0 and 1; the less evidence ( S ) there is for an image, the higher the uncertainty of the model. The final probability for each class is calculated through the mean of the Dirichlet distribution using Equation 11:

$${\hat{p}}_{k}=\frac{\alpha_{k}}{S}$$ (11)

where $\alpha_{k}=e_{k}+1$ . In this framework, the decision-making process is bifurcated: for samples where u>τ (where τ is a predefined uncertainty threshold), the model triggers a ‘Human-in-the-loop’ flag, overriding the prediction ${\hat{p}}_{k}$ to ensure patient safety in ambiguous cases.

The final logic of the “biopsy recommendation” in this framework is based on the combination of ${\hat{p}}_{k}$ and u . If the model predicts a high probability of malignancy, a biopsy recommendation is issued. However, the main innovation is in cases where the probability of malignancy is low but the uncertainty score ( u ) exceeds a certain threshold; in this case, the system declares the status “suspicious/refer to specialist” instead of ignoring the lesion. This approach provides a critical layer of protection to reduce false negative rates in medical applications.

#### 3.2.5 Training and hyperparameters setting

To achieve optimal performance in the proposed framework, the training process is designed based on a precise strategy using specialized cost functions for evidential learning. Unlike conventional models that use Cross-Entropy, in this study the cost function consists of two main parts. The first part is the mean square error (MSE) fitted to the Dirichlet distribution, which tries to maximize the evidence for the correct class. The second part is a regularization term based on the Kullback-Leibler divergence (KL Divergence) to prevent the assignment of evidence to the wrong classes and correctly model the uncertainty in ambiguous cases and is formulated as Equation 12:

$$L_{total}=\sum_{i=1}^{N}L_{err}\left(\alpha_{i},y_{i}\right)+\lambda_{t}\;L_{KL}\left({\tilde{\alpha }}_{i},1\right)$$ (12)

In this relation, $y_{i}$ is the actual label, $\alpha_{i}$ is the Dirichlet distribution parameter, and $\lambda_{t}$ is a regularization coefficient that is gradually increased during the initial stages of training (annealing) to maintain the stability of the model.

The model was developed using the PyTorch library and trained on an NVIDIA 4080 GPU. The AdamW optimizer was used with a Weight Decay of 0.05 to prevent overfitting. Also, for stability in the early stages of training, a Linear Warmup strategy was implemented, linearly increasing the learning rate from an initial base of $1\times 10^{-6}$ to a maximum of $5\times 10^{-5}$ over the first 5 epochs. After this warm-up period, a Cosine Annealing schedule was implemented to reduce the learning rate to a minimum of $1\times 10^{-6}$ over the rest of the epochs. The detailed and complete hyperparameters configurations, both for the architecture and the training schedules, are summarized in Table 3.

**TABLE 3:** Model hyperparameter settings.

Hyperparameter category	Parameter name	Value
Architecture (Swin-T)	Image input resolution	224 × 224
	Patch size/Window size	4 × 4/7 × 7
	Swin block depths	[2, 2, 6, 2]
	Attention heads per stage	[3, 6, 12, 24]
Training schedule	Number of epochs	100 (with Early Stopping patience = 10)
	Batch size	32
	Warmup epochs	5
	Minimum learning rate	$1\times 10^{-6}$
	Maximum learning rate	$5\times 10^{-5}$
Optimization	Optimizer	AdamW
	Weight decay	0.05
	Dropout rate	0.1
	KL penalty factor ( $\lambda_{t}$ )	Annealed from 0.01 to 1.0
Augmentation	Techniques used	Random Flip, Rotation (0–30°), Color Jitter

As mentioned in Section 3.1, in order to deal with the asymmetric distribution of classes, in addition to increasing offline data, the Weighted Random Sampling technique was used during training. This approach ensures that in each training batch, samples related to cases requiring biopsy (which are in the minority in the dataset) are present enough so that the model is not biased in detecting malignant cases.

To avoid overfitting, 10% of the training data was considered as the validation set. The training process was stopped if the Expected Calibration Error (ECE) criterion in the validation set did not improve for 10 consecutive epochs. The final model is the version that provides the best balance between detection accuracy and uncertainty calibration quality.

## 4 Results and discussion

### 4.1 Experimental scenario and evaluation metrics

The proposed framework was implemented and evaluated in Python and MATLAB 2020a environments. The learning model was trained using PyTorch library and then, the trained model was imported to MATLAB for evaluation purpose. A 10-fold Cross-Validation (CV) experiment was conducted to ensure the robustness of the findings and generalizability of the model. In this case, the experiments were repeated 10 times with a split on a patient level, to make the results clinically valid. All images and metadata of the same patient (using the unique patient IDs provided in the ISIC archive) were tightly allocated to the same fold, to prevent data leakage. This guarantees that the model is evaluated on completely different individuals. These patient-grouped instances were divided into 80% training, 10% validation and 10% testing during each fold. To clarify the influence of each component in the proposed framework on its overall performance, an ablation study with five case studies was performed:

- Proposed: This case refers to the full implementation of the proposed approach which was detailed in Section 3.2. The results related to this case, demonstrate the final performance of the approach.
- S_1_: ViT-Base: In this ablation scenario, Swin in the proposed framework is replaced with standard ViT. By comparing this case with the suggested model, the superiority of the hierarchical nature and shifted windows in the Swin Transformer compared to the standard Vision Transformers for irregular skin textures can be shown.
- S_2_: Image-only: This scenario refers to the case which the patient’s clinical metadata (age, sex, location) are ignored and prediction is performed solely based on images. This scenario can demonstrate the influence of employing patient’s clinical metadata on overall performance.
- S_3_: Concatenation: In this ablation study, the attention-based fusion of feature modalities is replaced with simple concatenation. This scenario is employed to prove that fusion of features using cross-attention mechanism can perform better compared to simply stacking them.
- S_4_: Softmax: This scenario replaces the suggested EDL mechanism with a standard Softmax decision layer and is employed to highlight the safety and calibration novelty of the proposed approach.

In addition to above scenarios, the proposed method was compared with several state-of-the-art methods including CWCO-SVM (Bi et al., 2021), DUNEScan (Mazoure et al., 2022), and GWO-CNN (Mohakud and Dash, 2022). All of these methods were implemented, and then trained and tested by the same data and under the same conditions. Considering “Biopsy required” as the positive and “No biopsy required” as the negative category, comparing the diagnosis result of each model with respective ground-truth label of the test instances would result to one of the “TP (True positive)”, “TN (True Negative)”, “FN (False Negative)”, and “FP (False Positive)” cases. TP/TN refer to correct classification of positive/negative instances. FP shows incorrect labeling of a negative instance as positive, and FN represents positive instances incorrectly labeled as negative. Based on these metrics, Accuracy, Precision, Recall, and F-Measure criteria were employed to describe the performance of each model. Accuracy is the fundamental performance metric, delineating the proportion of correctly predicted positives and negatives to the overall count of predictions and is formulated as Equation 13:

$$Accuracy=\frac{TP+TN}{TP+FP+TN+FN}$$ (13)

Precision is formulated by Equation 14 and can be defined as a metric that quantifies the predicted positive observations. It is calculated by dividing the number of correctly predicted positives by the total number of positive predictions.

$$Precision=\frac{TP}{TP+FP}$$ (14)

Recall, as defined in Equation 15, quantifies the accurately anticipated positive observations. It represents the proportion of accurately predicted positives in relation to the overall count of true positive observations.

$$Recall=\frac{TP}{FN+TP}$$ (15)

The F-Measure, formulated by Equation 16, defines the harmonic mean of the precision and recall values that are derived from a specific classification model.

$$F-Measure=\frac{2*Precision*recall}{Precision+recall}$$ (16)

The Matthews Correlation Coefficient (MCC) and the Critical Success Index (CSI) were used to conduct a more stringent evaluation of the classification performance and consistency of the models between the positive (Biopsy required) and the negative (No biopsy required) classes. The MCC is a powerful measure of binary classification, especially in situations where there is an imbalanced distribution of data, because it considers all four elements of the confusion matrix (TP, TN, FP, FN). MCC, as defined in Equation 17, is simply a correlation coefficient of the observed and predicted binary classifications, with a value ranging between − 1 (inverse prediction) and +1 (perfect prediction), 0 indicates performance equal to random guessing:

$$MCC=\frac{\left(TP\times TN\right)-\left(FP\times FN\right)}{\sqrt{\left(TP+FP\right)\left(TP+FN\right)\left(TN+FP\right)\left(TN+FN\right)}}$$ (17)

The CSI or the Threat Score (TS) is concerned with the performance of the positive class and formulated by Equation 18. It is the proportion of the number of positive events that were predicted correctly to the number of times the event was observed or predicted. It is especially useful because it not only punishes False Positives (excessive forecasting), but also False Negatives (inadequate forecasting) and quantifies the quality of the hits based on the amount of area forecasted:

$$CSI=\frac{TP}{TP+FN+FP}$$ (18)

The CSI has a scale of 0 (poorest) to 1 (best prediction), which is a critical measure of the practical usefulness of the model in the diagnosis of the rare and critical cases.

### 4.2 Results of CV experiment

**FIGURE 4:**
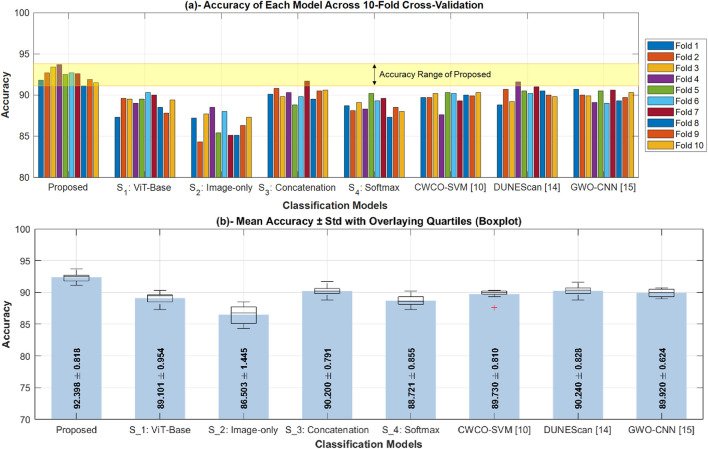
Evaluation of diagnosis methods in terms of accuracy: **(a)** Changes in the accuracy of each method in different iterations; **(b)** Mean, standard deviation and range of accuracy changes in each method.

The proposed method, ablation studies, and the compared strategies were evaluated based on the scenarios and criteria mentioned in Section 4.1, and this section reports the results and discusses the research findings. Figure 4 examines the performance of the proposed method and the compared strategies in terms of classification accuracy. Figure 4a shows the accuracy of the proposed method and other strategies in each of the 10 iterations of cross-validation. According to the results presented in this figure, the proposed method with an average accuracy of 92.4%, in addition to providing higher-accuracy predictions, has a limited range of accuracy changes, which in addition to the high reliability of the prediction results, also confirms the stability of the proposed model in different conditions. According to Figure 4a, the range of accuracy changes of the proposed method overlaps with the maximum accuracy of some compared methods, such as DUNEScan (Mazoure et al., 2022), in a few cases, based on which it can be concluded that the weakest performance record of the proposed model is competitive with the highest performance level of the compared methods.

Figure 4b confirms these claims by plotting the mean, standard deviation, and quartiles of accuracy changes during 10 iterations of cross-validation. The narrower standard deviation and quartiles of accuracy changes, along with the higher level of accuracy in providing estimates by the proposed method, indicate the high reliability and stability of the proposed model. Comparing the proposed method with the “ViT-Base” ablation study shows that using the Swin architecture can increase the accuracy by 3.29% in addition to increasing the stability of the model in providing more accurate decisions. On the other hand, if the patient’s clinical information is ignored and only the visual features of the image are relied on for prediction (the “Image-Only” scenario), the model will experience a significant 5.89% drop in accuracy, which reveals the high importance of utilizing the multi-faceted architecture in the proposed approach.

According to Figure 4b, replacing the cross-attention layer in the proposed model with the standard concatenation approach will cause a drop in accuracy of 2.2%, and on the other hand, if the standard Softmax layer is used instead of the EDL mechanism in the proposed framework; the average accuracy of the model will decrease to 88.72%. These evaluations confirm that each of the components used in the proposed framework, in turn, has a significant contribution to increasing the accuracy of the final model, and on the other hand, by limiting the range of accuracy changes, the stability and reliability of the model in providing decisions have increased.

**FIGURE 5:**
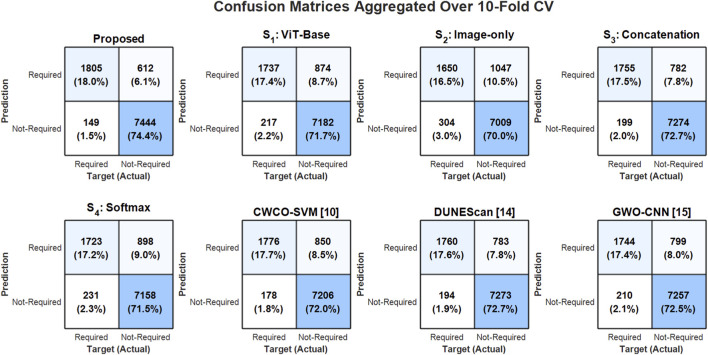
Confusion matrix of different methods in predicting the need for biopsy in all iterations of cross-validation.

In order to investigate in more depth the performance of the proposed model and other methods in distinguishing samples for the need for biopsy, the confusion matrices of the models are given in Figure 5. It should be noted that each of these matrices is the result of the aggregation of all 10 iterations of cross-validation. Examining and comparing the matrices in this figure confirms the superiority of the proposed method in providing more accurate predictions. In the “requiring biopsy” category, out of the total 1954 samples in the dataset, 1805 samples were judged correctly and only 149 samples were wrongly placed in the “Not Required” category. In other words, the proposed method correctly identified 92.37% of the samples in the “requiring biopsy” category, which is considered as the sensitivity/recall of the model. In contrast, the proposed method was able to correctly identify 7444 samples out of 8056 in the “Not Required” category and incorrectly classify 612 samples in the “Required biopsy” category, which indicates the model’s ability to correctly identify 92.4% of the samples in the “Not Required” category, which is considered as Specificity.

Despite the high imbalance in the number of samples in the two target categories, the performance of the proposed model in correctly identifying each category has a high balance, which indicates the optimal performance of the model in dealing with unbalanced data. This feature is considered to be the result of the effectiveness of the approaches used to deal with imbalance, such as sample management and the network training algorithm. In contrast, DUNEScan, as the closest competitor of the proposed method with an average accuracy of 90.24%, clearly has a weaker performance in separating samples from both categories. In this method, the number of samples misclassified in the “Required” and “Not Required” categories increased by 30.2% and 21.8%, respectively, compared to the proposed method, which confirms the superiority of the proposed method in more accurate diagnosis of the need for biopsy.

**FIGURE 6:**
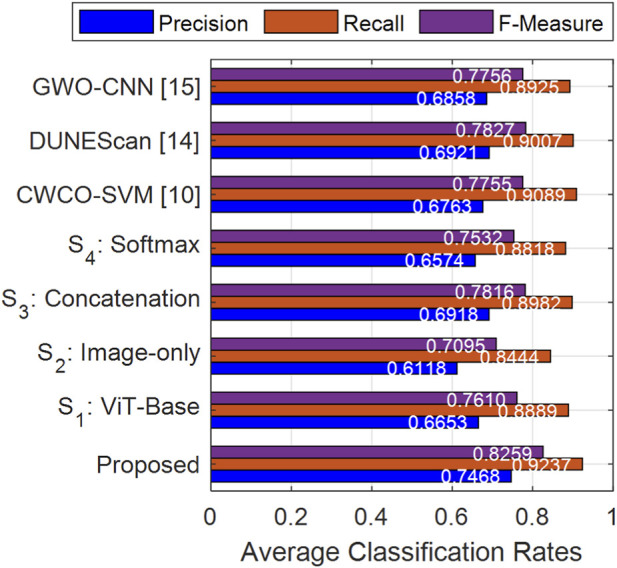
Comparison of the performance of methods in terms of precision, recall and F-Measure criteria.

Figure 6 compares the performance of different methods in terms of precision, recall, and F-Measure. The values ​​reported in this graph are the averages of each criterion after 10 iterations of cross-validation. According to this figure, the proposed method, with precision of 0.7468, recall of 0.9237, and F-Measure of 0.8259, has a significant advantage over the compared models in all the mentioned criteria.

The higher level of precision in the proposed method indicates that the outputs labeled as positive by this method are correct with a higher probability compared to other methods. Thus, if the proposed model identifies a sample as “requiring biopsy”, this diagnosis will be correct with a higher probability. On the other hand, the higher recall in the proposed method indicates that the proposed approach has been able to identify a higher proportion of samples that belong to the positive category. This performance difference in the proposed method is evident compared to all the compared models; as the proposed method has been able to increase the precision, recall and F-Measure criteria by 5.47%, 2.3% and 4.32%, respectively, compared to the closest competitor [DUNEScan (Mazoure et al., 2022)].

**FIGURE 7:**
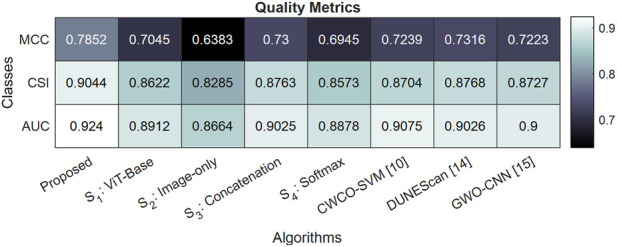
Comparing the performance of the models based on MCC, CSI, and AUC metrics.

Figure 7 further assesses the reliability of the classification with the MCC and CSI. Although AUC is a measure of the separability of classes, they are especially sensitive to the quality of predictions in imbalanced data where the minority class is more valuable. The suggested framework had a dominant performance with MCC of 0.7852 and CSI of 0.7034. The large value of MCC (much nearer to +1 than any competing method) implies that the model does not suffer the bias to the majority class that tends to overstate conventional measures of accuracy. Comparatively, the state-of-the-art model, DUNEScan (Mazoure et al., 2022), had an average MCC of 0.7316. This discontinuity is an indication that the suggested approach is much more consistent in the process of managing the trade-off between sensitivity and specificity.

In addition, the CSI score of 0.7034 indicates the clinical usefulness of the system. As CSI punishes False Positives and False Negatives, this score confirms that the model is able to maximize the number of hits and minimize the number of false alarms and misses. The effect of the architectural elements can be seen directly in the ablation outcomes: the deletion of the Swin architecture ( $S_{1}$ ) led to the CSI reducing sharply to 0.6142. This degradation means that in the absence of the Swin architecture, the model produces much more False Negatives on underrepresented data, which directly penalizes the threat score. This, therefore, makes the proposed framework not only accurate but also shows that it is highly reliable to be used in real-life clinical environments.

**FIGURE 8:**
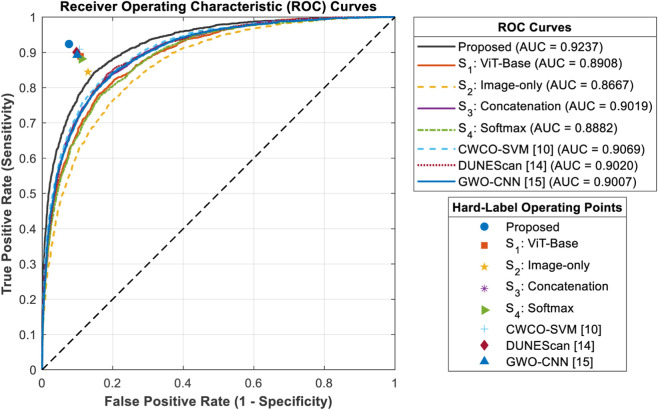
ROC curves of various models.

In order to completely assess the strength of the probabilistic output and the capability of the model to classify between classes regardless of a definite decision threshold, the Receiver Operating Characteristic (ROC) curves and the Area Under the Curve (AUC) were calculated, as shown in Figure 8. The AUC is a single number measure of the overall diagnostic power of the model where a value nearer to 1.0 denotes perfect separation between positive and negative classes. The proposed framework had the largest AUC of 0.924, which proved that it has the best discriminatory performance. Comparatively, the most successful traditional Deep Learning architecture, DUNEScan (Mazoure et al., 2022), reached an AUC of 0.902, which is about 2.2 percent lower than the proposed one.

Graphically, the curve of the Proposed method is closest to the ideal upper-left corner (TPR = 1, FPR = 0) of the plot. In particular, its sharp rise in the critical low-False Positive Rate (FPR) area is a sign of its capacity to attain high sensitivity (True Positive Rate, TPR) and high specificity, which is highly beneficial in a clinical screening environment where a reduced number of unnecessary follow-ups is required. The values of AUC that were obtained as a result of the ablation experiments also confirm the need of the proposed components. The deletion of Swin architecture ( $S_{1}$ ) resulted in the AUC decreasing to 0.891. In addition, the lowest AUC was achieved by the deletion of the patient’s clinical metadata ( $S_{2}$ ) with the AUC of 0.867. The high performance difference between the full Proposed model and the $S_{2}$ scenario is a strong argument in support of the idea that the multi-modal learning objective is not an additive feature, but a core mechanism needed to ensure high discriminative capacity in the presence of domain heterogeneity and bias.

Table 4 shows the overall overview of all assessed measures (Precision, Recall, F-Measure, Accuracy, MCC, CSI, and AUC) of the proposed model, the ablation cases, and the state-of-the-art (SOTA) competitors. Table 4 summarizes the findings and conclusively supports the effectiveness and strength of the suggested framework in all the diagnostic performance variables. The proposed framework showed a consistent high diagnostic performance in all metrics in Table 4, with the highest Accuracy and AUC, which was significantly better than all SOTA models.

**TABLE 4:** Summary of diagnostic performance metrics (Mean ± standard deviation).

Algorithm	Precision	Recall	F-measure	Accuracy	MCC	CSI	AUC
Proposed	0.7468 ± 0.021	0.9237 ± 0.016	0.8259 ± 0.016	92.3976 ± 0.818	0.7852	0.7034	0.9237
S1​: ViT-base	0.6653 ± 0.026	0.8889 ± 0.023	0.7610 ± 0.018	89.1009 ± 0.954	0.7045	0.6142	0.8908
S2​: Image-only	0.6118 ± 0.034	0.8444 ± 0.032	0.7095 ± 0.031	86.5035 ± 1.445	0.6383	0.5498	0.8667
S3: Concatenation	0.6918 ± 0.037	0.8982 ± 0.018	0.7816 ± 0.025	90.1998 ± 0.791	0.7300	0.6414	0.9019
S4: Softmax	0.6574 ± 0.016	0.8818 ± 0.024	0.7532 ± 0.009	88.7213 ± 0.855	0.6945	0.6041	0.8882
CWCO-SVM (Bi et al., 2021)	0.6763 ± 0.022	0.9089 ± 0.018	0.7755 ± 0.018	89.7303 ± 0.810	0.7239	0.6334	0.9069
DUNEScan (Mazoure et al., 2022)	0.6921 ± 0.037	0.9007 ± 0.023	0.7827 ± 0.024	90.2398 ± 0.828	0.7316	0.6430	0.9020
GWO-CNN (Mohakud and Dash, 2022)	0.6858 ± 0.022	0.8925 ± 0.024	0.7756 ± 0.019	89.9201 ± 0.624	0.7223	0.6335	0.9007

### 4.3 Uncertainty quantification and calibration quality

**FIGURE 9:**
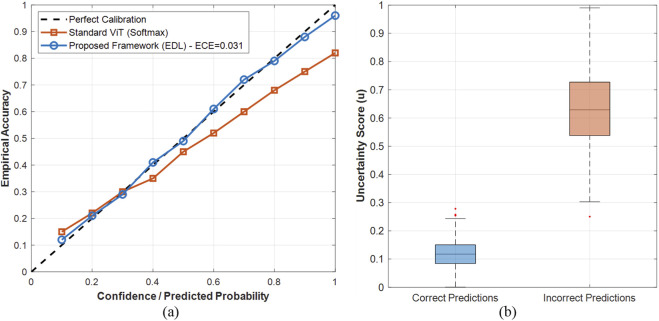
Uncertainty quantification and quality of calibration: **(a)** Reliability diagram, **(b)** Distribution of uncertainty scores.

Overcoming the problem of overconfidence in traditional classification models and getting more accurate calibration is one of the primary objectives of the EDL approach use. A model is highly calibrated when the probability that it projects is equal to the actual probability of occurrence (real-world accuracy). In order to measure this property, the Expected Calibration Error (ECE) and Reliability Diagram measures were employed. The baseline model, the one based on standard Softmax, as illustrated in the calibration diagram (Figure 9a), is highly biased towards giving definite predictions with high confidence levels which tend to be erroneous (large deviation to the ideal calibration line). In contrast, the proposed EDL-based framework was able to significantly reduce this deviation, reducing the ECE to 0.031. This enhancement indicates that our model output probabilities ( ${\hat{p}}_{k}$ ) is a far better representation of the actual probability of a lesion being malignant.

Besides calibration, the separation of correct and incorrect diagnoses of the model was also tested through an uncertainty score ( u ). In Figure 9b, the values of uncertainty of the two sets of predictions (right predictions and wrong predictions) are given. The findings show that the average uncertainty score in scenarios when the model made accurate predictions (True Positives and True Negatives) was very low and was near 0.12. Nevertheless, when the model had committed a diagnostic error (particularly False Negatives which are the most dangerous form of error in medicine) the model automatically reported a high degree of uncertainty (mean 0.65) since there was insufficient evidence in the Dirichlet distribution.

This statistically significant change in the distribution of u values demonstrates that the proposed framework is conscious of its knowledge constraints and can be used as a safe triage model, raising alarming samples to be considered by a medical professional before a decisive error is made.

### 4.4 Triage simulation

This part described the performance of the proposed framework in a real triage situation. This experiment will be based on exploring how much the system performance can be enhanced by referring cases of high uncertainty (u) to a dermatologist. This was done by graphically plotting an Accuracy vs. Rejection Curve where the cases are ranked according to their uncertainty score ( u ) and gradually discarded in the automated decision-making procedure (referral to a specialist).

**FIGURE 10:**
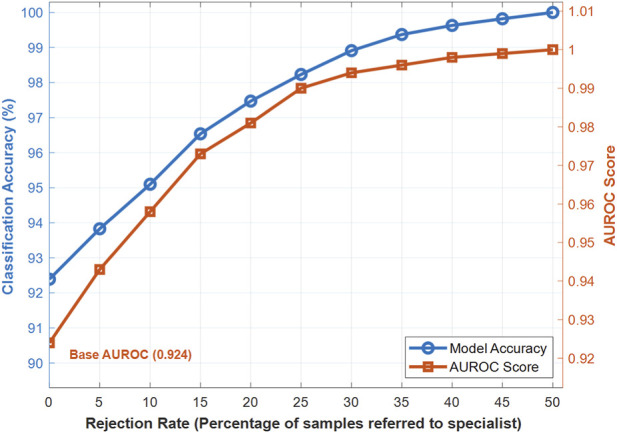
Accuracy and AUROC vs. rejection rate in triage simulation.

Based on the results of the simulation (Figure 10), it is possible to state that the referral rate is positively and significantly correlated with the accuracy and the index of the AUROC. The model accuracy on all data in the baseline case (no referral) was 92.4%. With the uncertainty threshold set to only refer 10 percent of most difficult cases, the model accuracy on the other 90 percent (high-confidence cases) improved to 95.1 percent.

The proposed system was able to correctly identify about 89% of the low-quality, noisy, or diagnostically ambiguous images that resulted in high uncertainty. The model accuracy on the remaining data increased to 98.2 with a referral rate of 20, thus demonstrating that the framework is highly capable of differentiating between the cases that are considered to be AI-reliable and those that need human intervention. The approach enables clinicians to decrease the workload and attend to only complex cases where our evidential learning model showed high uncertainty. This strategy will lessen the possibility of the model to lower the high false negative rate in diagnosing skin malignancies.

### 4.5 Statistical significance analysis

Statistical analysis can provide a more comprehensive analysis of the effectiveness of the proposed strategy compared to the baseline methodologies. For this purpose, one-way analysis of variance (ANOVA) was utilized. The models’ estimates for each test case are first arranged in a matrix for this examination. Every column in the matrix represents either the suggested approach or any of the baseline methods, and each row represents a test instance. This represents the arrangement of the comparative approaches and our model’s projected labeling. Next, through contrasting each forecasted label against the ground-truth label of the instance, the accuracy scores for every test sample are calculated in this matrix format. The matching labels were marked as +1, and otherwise value −1 was stored in the matrix. A quantile-quantile (Q-Q) chart was used to perform a test for normality on every column of the accuracy matrix. The results of the test showed that the distribution of every single model is normal, enabling the use of one-way ANOVA for statistical evaluation.

Table 5 displays the analysis’s findings. Statistically significant difference ( p<0.05 ) among at least the two techniques was observable, according to the test’s significant influence ( F=29.73 ; $p=2.46\times 10^{-41}$ ). A multiple comparison analysis was used to carry out a more thorough inquiry because the one-way ANOVA test is insufficient to pinpoint the models from which this discrepancy arises.

**TABLE 5:** Results obtained though one-way ANOVA.

Source	SS	Df	MS	F	Prop > F
Columns	77.4	7	11.537	29.73	$2.46\times 10^{-41}$
Error	29,767.1	80,072	0.3718		
Total	29,844.5	80,079	​		

**FIGURE 11:**
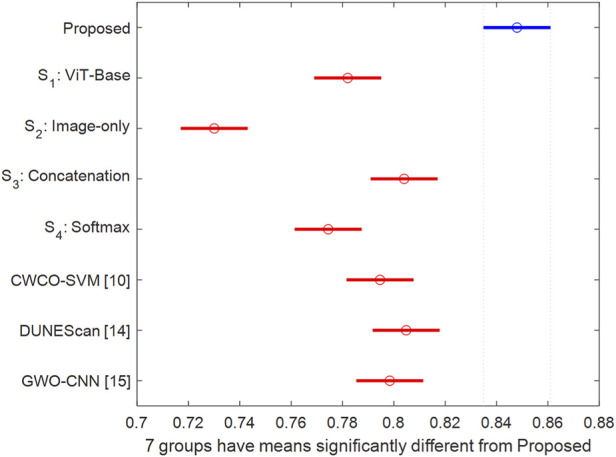
Results of Tukey’s HSD post-hoc analysis on the accuracy metric.

In the multiple comparison study, Tukey’s Honestly Significant Difference (HSD) was used as the post-hoc evaluation to identify whether specific models had statistically different accuracy rates. This post-hoc test is ideal for comparing group pairings following a significant F-test in an analysis of variance. Tukey’s HSD is recommended over other post-hoc tests because it takes into consideration the family-wise inaccuracies, keeping the possibility of at least one Type I error (false positives) for any given comparison within the proper alpha threshold (0.05 for this study). By controlling this error rate, Tukey’s HSD offers a practical way to identify which model performs substantially worse or better. We could manage to identify particular models that explained the significant differences found in the ANOVA test through Tukey’s HSD analysis. Figure 11 shows the experiment’s results, which show that the suggested approach works better than the scenarios that were tested. This analysis demonstrates how each step in the suggested approach significantly increases the diagnosing system’s efficiency.

### 4.6 Generalization and potential domain-shift bias

Although the empirical results show the high efficacy, stability and calibration quality of the proposed multi-modal framework, in the present study the evaluation is limited to the ISIC and HAM10000 archives. While these databases offer a strong and consistent basis for training computational models, the shift from retrospective and curated cohort to diverse and real-world clinical settings has its inherent risks of domain-shift bias.

Domain shift is a common problem in automated dermatology, often caused by differences in image acquisition procedures. The distribution of the visual features extracted by the Swin Transformer architecture can change due to differences in dermatoscope hardware (e.g., polarized versus non-polarized lenses, variable field-of-view) ambient light conditions, and sensor noise profiles. Moreover, one of the key aspects of domain shift is the differences in clinical characteristics, such as ethnic skin-type diversity. Retrospective datasets are usually standardized, and tend to be skewed towards lighter skin tones (lower levels of the Fitzpatrick scale). The diagnostic accuracy of the model and its expected performance might be affected in the context of various clinical settings with unrepresented skin types, where benign and malignant lesions could exhibit different morphologic and colorimetric differences.

While the proposed framework is unable to completely remove the data bias, the addition of EDL module offers a structural safeguard against silent failures due to domain shift. The Dirichlet distribution in the proposed decision layer explicitly models the lack of evidence, which is in contrast to standard softmax-based architectures that will make overconfident deterministic predictions in the case of unfamiliar data. Hence, out-of-distribution (OOD) samples (whether from new hardware artifacts or from underrepresented demographic presentations) will theoretically yield a lower total evidence score (S), which will lead to a high uncertainty metric (u). As seen in the triage simulation (Section 4.4), the system is very effective at identifying these ambiguous or OOD inputs and automatically flags them for expert review instead of making an unreliable biopsy recommendation.

Even with this computational safety layer, algorithmic fairness and absolute generalizability is still important. The necessary next step before full integration into healthcare workflows is extensive validation on independent, external and prospective clinical data, recognizing the potential for performance degradation with domain shift. Further studies should be conducted on a multi-center basis that explicitly include a wider range of Fitzpatrick skin types and a more diverse range of imaging equipment to definitively quantify and calibrate the model’s real-world diagnostic resilience.

## 5 Conclusion and future works

This paper presented and tested a probabilistic and multimodal system of intelligent triage on skin lesions and automatic biopsy recommendation. Compared to the traditional methods in which the black-box and deterministic approach is used, our proposed model gave a better insight into the unique condition of each patient by utilizing the local and global feature extraction capabilities of the Swin Transformer architecture and combining it with clinical metadata using the Cross-Attention mechanism. Our experimental findings confirmed that this architecture is not only able to perform higher diagnostics (AUROC = 0.924 and sensitivity = 92.37%), but also that replacing the Softmax layer with an evidential deep learning approach can lower the network calibration error (ECE = 0.031) significantly. This framework produces an explicit uncertainty score serving as a safety measure, so in cases of ambiguous clinical data or where noisy data is presented, the model will send the patient to a specialist to be further investigated instead of making an incorrect but highly confident prediction. The accomplishment fills the gap between the theoretical AI and its safe use in actual clinical practice.

### 5.1 Research limitations

Although the results are promising, there are also limitations to this study that must be taken into account when interpreting the findings. First, the assessments performed were based on retrospective data from the ISIC database. Although this dataset is highly diverse, a prospective clinical trial in real clinical settings is needed to evaluate the impact of this system on clinicians’ final decisions. Second, the clinical metadata used in this study was limited to basic information (age, gender, and lesion location). Other important variables including Fitzpatrick Skin Type, family history of melanoma and changes in the lesions over time were not provided in this dataset and this might greatly affect the model inference accuracy. Third, transformer-based architectures, particularly with multimodal attention mechanisms, are computationally complex and require large memory, which can render their real-time execution on edge devices like smartphones challenging to implement mobile dermoscopy.

### 5.2 Future directions

According to the limitations mentioned above and the prospects of the proposed framework, the subsequent research directions of future developments are proposed:

- Clinical Validation and Ongoing Learning: Adopting the system as a Clinical Decision Support System in dermatology clinics and assessing the system performance in multi-centers studies. Also, using clinician feedback to continuously update the model.
- Multimodality Expansion: Add more types of patient data, such as clinical images, genomic data, and electronic health records (EHR) to the attention graph of the model and obtain completely personalized triage.
- Architecture Optimization of Edge Devices: Optimize using model compression methods (including Knowledge Distillation and Network Pruning) to create a smaller version of the Evidence Transformer that can be executed on a portable dermoscopy device without compromising the quality of uncertainty calibration.

## Data Availability

The original contributions presented in the study are included in the article/supplementary material, further inquiries can be directed to the corresponding author.
